# Serum miRNAs Expression and SNAP-25 Genotype in Alzheimer’s Disease

**DOI:** 10.3389/fnagi.2019.00052

**Published:** 2019-03-11

**Authors:** Simone Agostini, Roberta Mancuso, Gaia Liuzzo, Elisabetta Bolognesi, Andrea Saul Costa, Anna Bianchi, Mario Clerici

**Affiliations:** ^1^IRCCS Fondazione Don Carlo Gnocchi, Milan, Italy; ^2^Department of Pathophysiology and Transplantation, University of Milan, Milan, Italy

**Keywords:** SNAP-25, microRNA, Alzheimer’s disease, mild cognitive impairment, genotyping, SNP

## Abstract

MicroRNAs (miRNAs) are small non-coding RNAs that control gene expression by binding their 3′ untranslated region (3′UTR) region; these molecules play a fundamental role in several pathologies, including Alzheimer’s disease (AD). Synaptosomal-associated protein of 25 kDa (SNAP-25) is a vesicular protein of soluble *N*-ethylmaleimide-sensitive factor attachment protein receptor (SNARE) involved in neural plasticity and in the exocytosis of neurotransmitters, processes that are altered in AD. Recent results showed that a reduction of SNAP-25 is associated with dementia, and that the rs363050 SNAP-25 polymorphism correlates with cognitive decline and brain atrophy, as well as with the outcome of multistructured rehabilitation in AD patients. We verified the presence of possible correlations between the serum concentration of miRNAs that bind the SNAP-25 3′UTR region and AD. Six different microRNAs (miR-181a-5p, miR-361-3p, miR-23a-3p, miR-15b-3p, 130a-3p and miR-27b-3p) that bind the SNAP-25 3′UTR region were measured by qPCR in serum of AD patients (*n* = 22), mild cognitive impairment (MCI) subjects (*n* = 22) and age- and sex-matched controls (*n* = 22); analysis of results was done stratified for the rs363050 SNAP-25 genotype. Results showed that miR-27b-3p, miR-23a-3p and miR181a-5p serum concentration was significantly reduced in rs363050 SNAP-25 GG homozygous AD patients. Notably, concentration of these miRNAs was comparable in rs363050 AA homozygous AD patients, MCI and healthy controls (HCs). Data herein suggest that miRNAs that bind the SNAP-25 3′UTR region interact with SNAP-25 polymorphisms to influence the neural plasticity typical of AD brains, possibly as a consequence of modulatory activity on SNAP-25 mRNA and/or protein.

## Introduction

Alzheimer’s disease (AD) is the most common age-related form of dementia (Alzheimer’s Association, 2014). The etiopathogenesis of the disease is still unknown, and AD is defined as a multifactorial pathology whose development and progression is the result of the combination of several factors (Carreiras et al., 2013). People with a cognitive decline which is more severe than expected, but without the pathological failure typical of AD, are diagnosed as being affected by mild cognitive impairment (MCI; Petersen, 2011; Forlenza et al., 2013).

Recent results suggested the involvment of synaptosomal-associated protein 25 (SNAP-25), a component of the soluble *N*-ethylmaleimide-sensitive factor attachment protein receptor (SNARE) complex involved in the exocytotic release of neurotransmitters during synaptic transmission (Antonucci et al., 2016), in neurological disorders, including AD (Noor and Zahid, 2017). Thus, it was shown that: (1) SNAP-25 protein expression is reduced in brains (Shimohama et al., 1997; Greber et al., 1999; Musunuri et al., 2016), and increased in cerebrospinal fluid (Brinkmalm et al., 2014; Sutphen et al., 2018) of AD patients compared to elderly healthy subjects; (2) the *SNAP-25* single nucleotide polymorphism (SNP) rs363050 is associated with alterations of categorical fluency and a reduced localized brain activity in AD patients (Guerini et al., 2014), and was shown to be predictive of a worst outcome in AD patients undergoing rehabilitative therapy (Guerini et al., 2016); (3) the overexpression of SNAP-25 in the rat adult dorsal hippocampus leads to the deregulation of memory consolidation (McKee et al., 2010); and (4) the overexpression of SNAP-25 in rat hippocampal neuron culture cells is associated with synaptic transmission disorders (Owe-Larsson et al., 1999).

MicroRNAs (miRNAs) are short non-coding RNAs (containing about 20–24 nucleotides) involved in mRNA silencing and post-transcriptional regulation of gene expression (Bartel, 2004) *via* their ability to bind the 3′untranslated region (3′UTR). miRNAs are thus key players in the normal function of cells, and impairments in their modulation, regulation and/or expression are associated with pathologies including tumors (Castro et al., 2017; Drusco and Croce, 2017; Elghoroury et al., 2018; Koutsaki et al., 2017; Mansoori et al., 2017), stroke (Vijayan and Reddy, 2016) and type 2 diabetes mellitus (Liang et al., 2018). A possible role for miRNAs alterations has been postulated in AD (Provost, 2010; Delay et al., 2012; Pan et al., 2016; Kumar et al., 2017; Wang et al., 2018). In the present pilot study, we verified whether serum concentration of the miRNAs that bind the 3′UTR region of *SNAP-25* differs when AD, MCI and healthy controls (HCs) individuals are compared and, if this was the case, whether the expression of these miRNAs is modulated by the *SNAP-25* rs363050 polymorphism.

## Materials and Methods

### Patients and Controls

The Discovery Cohort was formed by 12 elderly HCs who had already been genotyped for rs363050 *SNAP-25* (Guerini et al., 2016); six of them were homozygous for the G allele and six for the A allele.

For the Study Cohort 22 patients affected by AD and 22 individuals with a diagnosis of MCI were recruited by the Department of Neurorehabilitation of IRCCS Santa Maria Nascente, Don Carlo Gnocchi Foundation—ONLUS, Milan, Italy. Patients were diagnosed either with AD according to the NINCDS-ADRDA Work Group criteria (McKhann et al., 1984) or with MCI according to Petersen (Petersen, 2004) and Grundman operation criteria (Grundman et al., 2004). To exclude reversible cases of dementia, all patients underwent complete medical and neurological examination, laboratory analyses, and magnetic resonance imaging (MRI) or computed tomography (CT) scan. Twenty-two HCs matched with patients for gender and age were also enrolled in the study. All these 66 subjects (22 AD, 22 MCI and 22 HCs) had already been genotypized for rs363050 *SNAP-25* (Guerini et al., 2016), and were selected because they carried either the A or G homozygous allele for rs363050. No other complications, including malnutrition, vitamin deficiency syndromes or tumor pathologies, were present in any of the individuals enrolled in the study. Blood count, urine analysis, blood chemistry screen, serum folate, B12 levels, and thyroid function tests were normal in all the subjects.

This study was carried out in accordance with the recommendations of ethics committee of the Fondazione Don Carlo Gnocchi with written informed consent from all subjects, or their relatives when appropriate. All subjects gave written informed consent in accordance with the Declaration of Helsinki. The protocol was approved by the ethics committee of the Fondazione Don Carlo Gnocchi.

For the methodologies regarding *in silico* selection of miRNAs targeting *SNAP-25* 3′UTR, isolation of miRNAs from human serum samples, screening profile and validation of miRNAs targeting 3′UTR *SNAP-25*, genotyping, and statistical analysis, see Supplementary Material.

## Results

### Serum miRNAs Profiling: Discovery Cohort

Expression profile of 90 miRNAs targeting and putatively binding 3′UTR of *SNAP-25* that were selected by *in silico* analysis and are known to be expressed in serum was performed in all the individuals part of the Discovery Cohort (12 HCs; six rs363050 AA genotype vs. six rs363050 GG genotype). Fifty-seven of the 90 miRNAs were detected in serum. Serum relative concentration of six miRNAs was identified as being differentially modulated by the *SNAP-25* rs363050 genotype, as their expression ratio was >2.5-fold or <−2.5-fold in both software used (qbase and Qiagen Software): miR-181a-5p, miR-130a-3p, miR-15b-3p, miR27b-3p, miR-361-5p and miR-23a-3p; all these miRNAs were up regulated in GG genotype compared to AA genotype HC.

### Serum miRNAs Expression Validation: Study Cohort

Results were subsequently validated with single qPCR in a larger population (Study Cohort) that included AD, MCI and HC individuals (Table 1).

**Table 1:** Demographic, clinical data and laboratory findings of the study cohort.

	AD patients	MCI subjects	Healthy controls
**N**	22	22	22
**Gender (M:F)**	6:16	11:11	9:13
**Age, years**	78.00; 74.00–81.50	76.00; 72.00–80.00	70.00; 61.25–85.25
**MMSE**	22.15; 18.90–24.30	24.70; 22.63–27.68*	–
**ApoE ε-4 carriers (%)**	48	18	18
**miR-27b-3p (%)**	91	100	95
fold median; IQR	1.33; 0.35–3.70	1.74; 0.57–2.59	1.43; 0.40–2.29
**miR-130a-3p (%)**	36	55	36
fold median; IQR	0.79; 0.08–3.14	1.25; 0.24–2.68	1.04; 0.14–1.94
**miR-15b-3p (%)**	14	32	18
fold median; IQR	0.95; 0.48–1.82	0.82; 0.34–1.76	0.77; 0.49–2.54
**miR-23a-3p (%)**	100	100	100
fold median; IQR	0.90; 0.20–3.39	1.32; 0.49–1.99	0.95; 0.44–2.81
**miR-181a-5p (%)**	65^#^	95	77
fold median; IQR	1.90; 1.20–3.20	1.64; 0.80–4.33	0.96; 0.62–1.89
**miR-361–3p (%)**	59	100	77
fold median; IQR	0.75; 0.23–5.72	1.25; 0.77–2.43	1.05; 0.55–4.35

Data are reported as median and interquartile range. AD, Alzheimer’s disease; MCI, mild cognitive impairment; M, male; F, female; MMSE, mini mental state evaluation; ApoE, apolipoprotein E; IQR, interquartile range. *p = 0.01 between AD and MCI subjects. ^#^*p* = 0.02 between AD and MCI subjects.

miR-23a-3p was detected in all individuals whereas miR-15b-3p was observed only in few cases (AD: 14%; MCI: 32%; HCs: 18%). miR-181a-5p, was statistically more frequent in MCI (95%) compared to AD patients (65%; *p* = 0.02), whereas HCs had an intermediate frequency (77%). Frequency and fold expression of each miRNA is summarized in Table 1.

GG homozygous rs363050 genotype AD patients (ADGG) were characterized by a lower expression of miR-27b-3p, miR-23a-3p and miR181a-5p compared to HCs, regardless of the rs363050 genotypes, as well as to all other groups.

**Figure 1:**
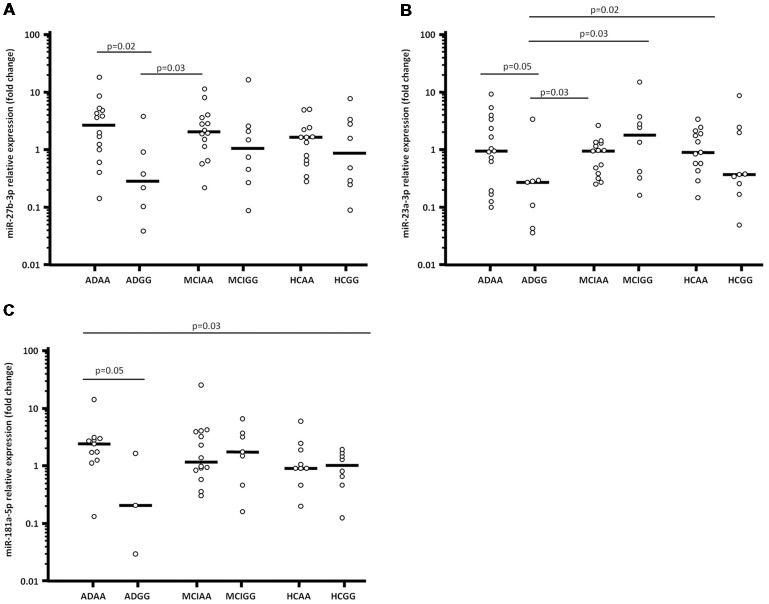
miR-27b-3p **(A)**, miR-23a-3p **(B)** and miR-181a-5p **(C)** expression fold (Log_10_ transformed) in serum of Alzheimer’s disease (AD) patients, mild cognitive impairment (MCI) individuals and healthy controls (HCs) splitted forsynaptosomal-associated protein of 25 kDa (*SNAP-25*) rs363050 genotype. ADAA, AA homozygous AD patients; ADGG, GG homozygous AD patients; MCIAA, AA homozygous MCI individuals; MCIGG, GG homozygous MCI individuals; HCAA, AA homozygous HCs; HCGG, GG homozygous HCs.

In particular, ADGG were characterized by a significantly lower miR-27b-3p expression (0.23; 0.08–0.71), compared to in AA homozygous AD (ADAA; 2.23; 0.79–3.84; *p* = 0.02) and MCI individuals (MCIAA; 1.63; 0.89–2.89; *p* = 0.03; Figure 1A).

Moreover, miR-23a-3p expression reduced as well in ADGG (0.27; 0.05–0.29) compared to all the other groups; this reduction reached statistical significance in comparison with AA homozygous HCs (HCAA; 0.97; 0.56–2.23; *p* = 0.02), ADAA (1.03; 0.30–3.65; *p* = 0.05), MCIAA (1.04; 0.39–1.42; *p* = 0.03), and GG homozygous MCI individuals (MCIGG; 2.14; 0.38–1.01; *p* = 0.03; Figure 1B).

Finally, miR-181a-5p expression was lower in ADGG (0.17; 0.02–1.56) compared to all the other groups (vs. ADAA: 2.33; 1.27–2.87; *p* = 0.05; Figure 1C). Overall results of miRNAs expression considering the rs363050 genotype are summarized in Supplementary Table S1. No differences were found in relation with sex, apolipoprotein E (*ApoE*) genotype or mini mental state evaluation (MMSE) score.

## Discussion

AD is a neurodegenerative disorder in which the rate of decline and functional restoration depend on the capacity for neural plasticity within residual neural tissues (Goldberg et al., 2015); this is at least partially influenced by polymorphisms in genes involved in synaptic transmission, including *SNAP-25* (Guerini et al., 2014, 2016). In particular, the rs363050 SNP of *SNAP-25* was shown to correlate with brain activity (Guerini et al., 2014) and the outcome of rehabilitation (Guerini et al., 2016) in AD patients.

By binding the 3′UTR region of genes, miRNAs inhibit the translation of mRNA into proteins, thus modulating gene expression (Cai et al., 2009). All the miRNAs analyzed in the present work putatively target and modulate *SNAP-25* expression, although miR-27a/b alone has so far been shown to inhibit the activity of *SNAP-25* (Machitani et al., 2017). Herein, we describe that the serum expression of miR-27b-3p, miR-23a-3p and miR-181a-5p is significantly reduced in AD patients that are homozygous for the G allele in the rs363050 SNP of *SNAP-25*. Although it is expressed in an intronic region, rs363050 was shown to modulate the expression of SNAP-25 protein; GG homozygosity, in particular, results in a reduced production of the protein compared to the AA homozygosis (Braida et al., 2015). Importantly, three independent studies detected a very low expression of SNAP-25 protein in the brain of AD patients compared to those of elderly not-AD individuals (Shimohama et al., 1997; Greber et al., 1999; Musunuri et al., 2016). Interestingly, miRNAs miR-27b-3p, miR-23a-3p and miR-181a-5p were also recently shown to be deregulated in serum of patients that underwent ischemic stroke (Wu et al., 2017; Cheng et al., 2018; Vijayan et al., 2018).

Based on these results it is tempting to speculate that in rs363050 GG homozygous AD patients, in whom SNAP-25 protein expression is reduced because of their genetic background, lower amounts of miRNAs that down regulate SNAP-25 expression would be an attempt to bypass such reduced SNAP-25 levels. In MCI individuals this phenomenon is not present possibly because modulation of miRNA expression only characterizes the later phases of dementia.

It is important to note that, as SNAP-25 is more expressed in excitatory neurons than in inhibitory neurons (Frassoni et al., 2005; Garbelli et al., 2008), these miRNAs could have neuroprotective properties; this possibility needs to be analyzed more in-depth.

It is nevertheless important to underline that we analyzed miRNAs expression in serum alone; these data might not reflect what goes on intracellularly, where miRNA-mRNA interactions, and the consequent modulation of gene expression take place. For these reasons our results need to be validated in a larger cohort, including also rs363050 heterozygous subjects, in which intracellular miRNA expression will need to be investigated. Finally, further experiment based on luciferase assay will be necessary to confirm the interaction between the miRNAs and SNAP-25 3′ UTR region and the effects of such interaction on SNAP-25 expression.

In conclusion, results of this pilot study, although preliminary and requiring to be confirmed by other independent studies with larger cohorts, suggest that the interaction between genetic and epigenetic factors modulate SNAP-25 expression and could contribute to the alterations in synaptic functionality, activity, neuro-plasticity observed in AD.

## Data Availability

The raw data supporting the conclusions of this manuscript will be made available by the corresponding author, without undue reservation, to any qualified researcher upon request.

## Author Contributions

SA and MC designed the experiments and analyzed the data. AB enrolled the subjects and collected the biological samples. SA, GL, EB and AC performed the experiments and data collection. SA, RM and MC interpreted the data and drafted the manuscript.

## Conflict of Interest Statement

The authors declare that the research was conducted in the absence of any commercial or financial relationships that could be construed as a potential conflict of interest.

## References

[B1] Alzheimer’s Association. (2014). 2014 Alzheimer’s disease facts and figures. Alzheimers Dement. 10, e47–e92. 10.1016/j.jalz.2014.02.001 24818261

[B2] Antonucci F. Corradini I. Fossati G. Tomasoni R. Menna E. Matteoli M. (2016). SNAP-25, a known presynaptic protein with emerging postsynaptic functions. Front. Synaptic Neurosci. 8:7. 10.3389/fnsyn.2016.00007 27047369 PMC4805587

[B3] Bartel D. P. (2004). MicroRNAs: genomics, biogenesis, mechanism, and function. Cell 116, 281–297. 10.1016/S0092-8674(04)00045-5 14744438

[B4] Braida D. Guerini F. R. Ponzoni L. Corradini I. De Astis S. Pattini L.. (2015). Association between SNAP-25 gene polymorphisms and cognition in autism: functional consequences and potential therapeutic strategies. Transl. Psychiatry 5:e500. 10.1038/tp.2014.136 25629685 PMC4312830

[B5] Brinkmalm A. Brinkmalm G. Honer W. G. Frölich L. Hausner L. Minthon L.. (2014). SNAP-25 is a promising novel cerebrospinal fluid biomarker for synapse degeneration in Alzheimer’s disease. Mol. Neurodegener. 9:53. 10.1186/1750-1326-9-53 25418885 PMC4253625

[B6] Cai Y. Yu X. Hu S. Yu J. (2009). A brief review on the mechanisms of miRA regulation. Genomics Proteomics Bioinformatics 7, 147–154. 10.1016/S1672-0229(08)60044-3 20172487 PMC5054406

[B7] Carreiras M. C. Mendes E. Perry M. J. Francisco A. P. Marco-Contelles J. (2013). The multifactorial nature of Alzheimer’s disease for developing potential therapeutics. Curr. Top. Med. Chem. 13, 1745–1770. 10.2174/15680266113139990135 23931435

[B8] Castro D. Moreira M. Gouveia A. M. Pozza D. H. De Mello R. A. (2017). MicroRNAs in lung cancer. Oncotarget 8, 81679–81685. 10.18632/oncotarget.20955 29113423 PMC5655318

[B9] Cheng X. Kan P. Ma Z. Wang Y. Song W. Huang C.. (2018). Exploring the potential value of miR-148b-3p, miR-151b and miR-27b-3p as biomarkers in acute ischemic stroke. Biosci. Rep. 38:BSR20181033. 10.1042/bsr20181033 30361294 PMC6259016

[B10] Delay C. Mandemakers W. Hébert S. S. (2012). MicroRNAs in Alzheimer’s disease. Neurobiol. Dis. 46, 285–290. 10.1016/j.nbd.2012.01.003 22285895

[B11] Drusco A. Croce C. M. (2017). MicroRNAs and cancer: a long story for short RNAs. Adv. Cancer Res. 135, 1–24. 10.1016/bs.acr.2017.06.005 28882219

[B12] Elghoroury E. A. ElDine H. G. Kamel S. A. Abdelrahman A. H. Mohammed A. Kamel M. M.. (2018). Evaluation of miRNA-21 and miRNA Let-7 as prognostic markers in patients with breast cancer. Clin. Breast Cancer 18, e721–e726. 10.1016/j.clbc.2017.11.022 29292183

[B13] Forlenza O. V. Diniz B. S. Stella F. Teixeira A. L. Gattaz W. F. (2013). Mild cognitive impairment. Part 1: clinical characteristics and predictors of dementia. Braz. J. Psychiatry 35, 178–185. 10.1590/1516-4446-2012-3503 23904025

[B14] Frassoni C. Inverardi F. Coco S. Ortino B. Grumelli C. Pozzi D.. (2005). Analysis of SNAP-25 immunoreactivity in hippocampal inhibitory neurons during development in culture and *in situ*. Neurosicence 131, 813–823. 10.1016/j.neuroscience.2004.11.042 15749336

[B15] Garbelli R. Inverardi F. Medici V. Amadeo A. Verderio C. Matteoli M.. (2008). Heterogeneous expression of SNAP-25 in rat and human brain. J. Comp. Neurol. 506, 373–386. 10.1002/cne.21505 18041776

[B16] Goldberg A. Curtis C. L. Kleim J. A. (2015). Linking genes to neurological clinical practice: the genomic basis for Neurorehabilitation. J. Neurol. Phys. Ther. 39, 52–61. 10.1097/npt.0000000000000066 25415554

[B17] Greber S. Lubec G. Cairns N. Fountoulakis M. (1999). Decreased levels of synaptosomal associated protein 25 in the brain of patients with Down syndrome and Alzheimer’s disease. Electrophoresis 20, 928–934. 10.1002/(sici)1522-2683(19990101)20:4/5<928::aid-elps928>3.3.co;2-q 10344268

[B18] Grundman M. Petersen R. C. Ferris S. H. Thomas R. G. Aisen P. S. Bennett D. A.. (2004). Mild cognitive impairment can be distinguished from Alzheimer disease and normal aging for clinical trials. Arch. Neurol. 61, 59–66. 10.1001/archneur.61.1.59 14732621

[B19] Guerini F. R. Agliardi C. Sironi M. Arosio B. Calabrese E. Zanzottera M.. (2014). Possible association between SNAP-25 single nucleotide polymorphisms and alterations of categorical fluency and functional MRI parameters in Alzheimer’s disease. J. Alzheimers Dis. 42, 1015–1028. 10.3233/jad-140057 25024311

[B20] Guerini F. R. Farina E. Costa A. S. Baglio F. Saibene F. L. Margaritella N.. (2016). ApoE and SNAP-25 polymorphisms predict the outcome of multi dimensional stimulation therapy rehabilitation in Alzheimer’s disease. Neurorehabil. Neural Repair 30, 889–893. 10.1177/1545968316642523 27075583

[B21] Koutsaki M. Libra M. Spandidos D. A. Zaravinos A. (2017). The miR-200 family in ovarian cancer. Oncotarget 8, 66629–66640. 10.18632/oncotarget.18343 29029543 PMC5630443

[B22] Kumar S. Vijayan M. Reddy P. H. (2017). MicroRNA-455–3p as a potential peripheral biomarker for Alzheimer’s disease. Hum. Mol. Genet. 26, 3808–3822. 10.1093/hmg/ddx267 28934394 PMC6075184

[B23] Liang Y. Z. Li J. J. Xiao H. B. He Y. Zhang L. Yan Y. X. (2018). Identification of stress-related microRNA biomarkers in type 2 diabetes mellitus: a systematic review and meta-analysis. J. Diabetes. [Epub ahead of print]. 10.1111/1753-0407.12643 29341487

[B24] Machitani M. Sakurai F. Wakabayashi K. Nakatani K. Tachibana M. Mizuguchi H. (2017). MicroRNA miR-27 inhibits adenovirus infection by suppressing the expression of SNAP25 and TXN2. J. Virol. 91:e00159-17. 10.1128/jvi.00159-17 28356525 PMC5446650

[B25] Mansoori B. Mohammadi A. Shirjang S. Baradaran B. (2017). MicroRNAs in the diagnosis and treatment of cancer. Immunol. Invest. 46, 880–897. 10.1080/08820139.2017.1377407 29058545

[B26] McKee A. G. Loscher J. S. O’Sullivan N. C. Chadderton N. Palfi A. Batti L.. (2010). AAV-mediated chronic over-expression of SNAP-25 in adult rat dorsal hippocampus impairs memory-associated synaptic plasticity. J. Neurochem. 112, 991–1004. 10.1111/j.1471-4159.2009.06516.x 20002519

[B27] McKhann G. Drachman D. Folstein M. Katzman R. Price D. Stadlan E. M. (1984). Clinical diagnosis of Alzheimer’s disease: report of the NINCDS-ADRDA Work Group under the auspices of Department of Health and Human Services Task Force on Alzheimer’s disease. Neurology 34, 939–944. 10.1212/wnl.34.7.939 6610841

[B28] Musunuri S. Khoonsari P. E. Mikus M. Wetterhall M. Häggmark-Mänberg A. Lannfelt L.. (2016). Increased levels of extracellular microvesicle markers and decreased levels of endocytic/exocytic proteins in the Alzheimer’s disease brain. J. Alzheimers Dis. 54, 1671–1686. 10.3233/jad-160271 27636840

[B29] Noor A. Zahid S. (2017). A review of the role of synaptosomal-associated protein 25 (SNAP-25) in neurological disorders. Int. J. Neurosci. 127, 805–811. 10.1080/00207454.2016.1248240 27734716

[B30] Owe-Larsson B. Berglund M. Kristensson K. Garoff H. Larhammar D. Brodin L.. (1999). Perturbation of the synaptic release machinery in hippocampal neurons by overexpression of SNAP-25 with the Semliki Forest virus vector. Eur. J. Neurosci. 11, 1981–1987. 10.1046/j.1460-9568.1999.00614.x 10336667

[B31] Pan Y. Liu R. Terpstra E. Wang Y. Qiao F. Wang J.. (2016). Dysregulation and diagnostic potential of microRNA in Alzheimer’s disease. J. Alzheimers Dis. 49, 1–12. 10.3233/jad-150451 26484912

[B32] Petersen R. C. (2004). Mild cognitive impairment as a diagnostic entity. J. Intern. Med. 256, 183–194. 10.1111/j.1365-2796.2004.01388.x 15324362

[B33] Petersen R. C. (2011). Clinical practice. N. Engl. J. Med. 364, 2227–2234. 10.1056/NEJMcp0910237 21651394

[B34] Provost P. (2010). MicroRNAs a molecular basis for mental retardation, Alzheimer’s and prion diseases. Brain Res. 1338, 58–66. 10.1016/j.brainres.2010.03.069 20347722 PMC2896967

[B35] Shimohama S. Kamiya S. Taniguchi T. Akagawa K. Kimura J. (1997). Differential involvement of synaptic vesicle and presynaptic plasma membrane proteins in Alzheimer’s disease. Biochem. Biophys. Res. Commun. 236, 239–242. 10.1006/bbrc.1997.6940 9240416

[B36] Sutphen C. L. McCue L. Herries E. M. Xiong C. Landenson J. H. Holtzman D. M.. (2018). Longitudinal decreases in multiple cerebrospinal fluid biomarkers of neuronal injury in synamptomatic late onset alzheimer’s disease. Alzheimers Dement. 14, 869–879. 10.1016/j.jalz.2018.01.012 29580670 PMC6110083

[B38] Vijayan M. Kumar S. Yin X. Zafer D. Chanana V. Cengiz P.. (2018). Identification of novel circulatory microRNA signatures linked to patients with ischemic stroke. Hum. Mol. Genet. 27, 2318–2329. 10.1093/hmg/ddy136 29701837 PMC6005038

[B37] Vijayan M. Reddy P. H. (2016). Peripheral biomarkers of stroke: focus on circulatory microRNAs. Biochim. Biophys. Acta 1862, 1984–1993. 10.1016/j.bbadis.2016.08.003 27503360 PMC5343760

[B39] Wang X. Liu D. Huang H. Z. Wang Z. H. Hou T. Y. Yang X.. (2018). A novel microRNA-124/PTPN1 signal pathway mediates synaptic and memory deficits in Alzheimer’s disease. Biol. Psychiatry 83, 395–405. 10.1016/j.biopsych.2017.07.023 28965984

[B40] Wu J. Fan C. L. Ma L. J. Liu T. Wang C. Song J. X.. (2017). Distinctive expression signatures of serum microRNAs in ischaemic stroke and transient ischaemic attack patients. Thromb. Haemost. 117, 992–1001. 10.1160/TH16-08-0606 28251236

