# Supplementary material for: Serum miRNAs Expression and SNAP-25 Genotype in Alzheimer’s Disease
Source: Front Aging Neurosci. 2019 Mar 11;11:52. doi: 10.3389/fnagi.2019.00052 (PMC6421304; doi:10.3389/fnagi.2019.00052)
Supplement: Supplementary file 1 [file Table_1.docx]

**Serum miRNAs expression and SNAP-25 genotype in
Alzheimer’s Disease**

Simone Agostini^1*^, Roberta Mancuso^1^, Gaia Liuzzo^1^, Elisabetta Bolognesi^1^, Andrea Saul Costa^1^, Anna Bianchi^1^, Mario Clerici^1,2^

^1^IRCCS Fondazione Don Carlo Gnocchi, Milan, Italy.

^2^Department of Pathophysiology and Transplantation, University of Milan, Milan, Italy.

**Supplementary Material**

**Material and Methods**

***In silico* selection of miRNAs targeting *SNAP-25* 3’UTR and screening in human serum**

miRNAs targeting *SNAP-25* 3’UTR were predicted *in silico* using three different bioinformatics databases: TargetScan (<http://www.targetscan.org/vert_71/>), microRNA.org (<http://www.microrna.org/microrna/home.do>) and miRdSNP (<http://mirdsnp.ccr.buffalo.edu/>).

**miRNAs isolation from human serum samples and cDNA reverse transcription**

miRNA isolation from serum was performed with a column-based kit (MiRNeasy serum/plasma Kit, Qiagen GmbH, Hilden, Germany), according to manufacter’s specific protocol. For samples of Discovery Cohort, 1 μl of synthetic UniSP2, UniSP4, UniSP5 and 5 UniSP6, and 5 μl of synthetic UniSP6 and miR-39 (*C. elegans*, *C.el.-*miR-39-3p) (5 nM), were added to 200 μl of serum after denaturation with Qiazol Lysis Reagent, whereas for samples of Study Cohort, just 5 μl of *C.el.-*miR-39-3p were added.

Total RNA was eluted in 10 μl. Two μl of RNA were utilized for reverse transcription reactions (in a final volume of 10 μl), performed in triplicate using the universal cDNA synthesis kit (miRCURY LNA^TM^ Universal cDNA synthesis kit, Exiqon Inc., Vedbaek, Denmark). To avoid variation due to sample differences and handling, all the variable involved in the procedure were kept consistent throughout the study.

**Screening of miRNAs targeting 3’UTR *SNAP-25***

The miRNAs targeting the 3’UTR region of *SNAP-25*, selected by *in silico* analysis, were included in a custom-designed miRCURY LNA^TM^ Universal RT microRNA PCR array (Pick&mix RT-QPCR plate, Exiqon Inc). These arrays included wells to check isolation (UniSp2, UniSp4 and UniSp5) and reverse transcription (UniSP6 and *C.el.-*miR-39-3p) efficiencies (UniSp2, UniSp4 and UniSp5 and UniSp6), as well as positive and negative PCR control, and an “interplate calibrator” (UniSp3). Ten μl of cDNA (50x diluted) were put in each wells, and the qPCR experiments were run in CFX96Touch real-time PCR Detection System (Bio-Rad, Hercules CA, US).

**Measurement of selected circulatory miRNA**

Specific LNA^TM^-individual microRNAs assays (Exiqon Inc.) were utilized to detect in sera the selected miRNA and reference miRNA (cel*-*miR-39-3p, cat. 203952, Exiqon Inc.) to normalize the results, according to the manufacturer’s instructions and as previously described (Mancuso et al., 2015).

Briefly, qPCR was performed on real time PCR system (CFX96Touch real-time PCR Detection System, BioRad, Hercules, CA, US) in 10 μl of reaction mix containing SYBR GREEN master mix (Exiqon Inc.), specific primer set for each miRNA and 4 μl of cDNA (40x diluted). Each cDNA template was tested in triplicate by qPCR. Negative controls, without rt-template controls, and no-template controls were included in each session. An additional step in the qPCR analysis was performed to evaluate the specificity of the amplification products by generating a melting curve for each reaction.

***ApoE* and *SNAP-25* genotyping**

Genomic DNA was isolated from whole blood by phenol-chloroform extraction. Customer-design Taqman probes for the 112 and 158 codons were used to determine the genotype of *apoliprotein E* gene (*ApoE*) (Costa et al., 2017), whereas the Taqman SNP Genotyping Assay (Life Technologies, Foster City, CA, US) was used to type SNP rs363050 of *SNAP-25* (Guerini et al., 2016).

**Data processing and statistical analysis**

For qPCR arrays, all the synthetic miRNAs were used for normalization. Baseline and threshold were manually set on the instrument for the evaluation of row Cq value for each sample. Because of the scarcity of miRNA serum, Cq=40 was set as the cut-off. Relative quantification was determined by the ΔΔCt method with normalization of the raw data to reference miRNA (Fleige et al., 2006). Data obtained from array experiments were analyzed by two different software: PCR Array Data Analysis of Qiagen (Qiagen) and qbase+ (BioGazelle, Ghent, Belgium). Statistical analyses were accomplished using commercial software (MedCalc, version 11.5.0.0). Demographic and clinical data were normally distributed and reported as mean ± standard deviation. The other quantitative variables, not normally distributed, were expressed as median and 25th-75th percentile. Logarithmic transformation was applied to miRNAs relative expression fold, and Kruskal-Wallis was used to compare value among groups, whereas Mann Whitney test was used to determine the significance between two groups. p values corresponding to <0.05 are described as statistically significant in the text. Considering a posteriori our data, the power for our sample size is 0.78.

**Table S1.** miRNAs fold expression in subjects of Study Cohort considering rs363050 genotype

| n | **AD patients**  7 15 | | **MCI subjects**  8 14 | | **HC subjects**  9 13 | |
| --- | --- | --- | --- | --- | --- | --- |
|  | **GG** | **AA** | **GG** | **AA** | **GG** | **AA** |
| **miR-27b-3p** | 0.23; 0.08-0.71 | 2.23; 0.79-3.84* | 0.87; 0.28-1.85 | 1.63; 0.89-2.89* | 0.81; 0.21-2.45 | 1.28; 0.49-1.79 |
| **miR-130a-3p** | 1.06; 0.79-2.96 | 0.09; 0.6-1.68 | 0.35; 0.09-1.34 | 1.19; 0.22-3.04 | 0.11; 0.07-2.87 | 1.13; 0.70-1.46 |
| **miR-15b-3p** | --- | 0.74; 0.37-1.41 | 1.45# | 0.47; 0.25-1.08 | 0.36# | 0.79; 0.49-2.55 |
| **miR-23a-3p** | 0.27; 0.05-0.29 | 1.03; 0.30-3.65* | 2.14; 0.38-1.01* | 1.04; 0.39-1.42* | 0.38; 0.23-2.38 | 0.97; 0.56-2.23* |
| **miR-181a-5p** | 0.17; 0.02-1.56 | 2.33; 1.27-2.87* | 1.65; 0.66-3.55 | 1.09; 0.75-3.94 | 0.96; 0.49-1.48* | 0.83; 0.72-1.94 |
| **miR-361-3p** | 0.41; 0.15-2.09 | 0.45; 0.11-3.48 | 0.71; 0.15-1.39 | 0.68; 0.46-1.25 | 0.35; 0.07-0.54 | 1.52; 0.52-3.20 |

AD: Alzheimer’s Disease

MCI: Mild Cognitive Impairment

HC: Healthy Controls

Data are expressed as median and Interquartile Range.

*p<0.05 compared to ADGG

#miRNA was found only in one subject

**REFERENCES**

Costa, A.S., Agostini, S., Guerini, F.R., Mancuso, R., Zanzottera, M., Ripamonti, E., et al. (2017). Modulation of immune responses to herpes simplex virus type 1 by IFNL3 and IRF7 polymorphisms: a study in Alzheimer’s disease. J. Alzheimers. Dis. 60, 1055-1063. doi: 10.3233/JAD-170520.

Fleige, S., Walf, V., Huch, S., Prgomet, C., Sehm, J., and Pfaffl, M.W. (2006). Comparison of relative mRNA quantification models and the impact of RNA integrity in quantitative real-time RT-PCR. Biotechnol. Lett. 28, 1601-1613.

Guerini, F.R., Farina, E., Costa, A.S., Baglio, F., Saibene, F.L., Margaritella, N., et al. (2016). ApoE and SNAP-25 polymorphisms predict the outcome of multi dimensional stimulation therapy rehabilitation in Alzheimer’s disease. Neurorehabil. Neural. Repair. 30, 889-893. doi: https://doi.org/10.1177/1545968316642523

Mancuso, R., Hernis, A., Agostini, S., Rovaris, M., Caputo, D., and Clerici, M. (2015). MicroRNA-572 expression in multiple sclerosis patients with different patterns of clinical progression. J. Transl. Med. 13, 148. doi: 10.1186/s12967-015-0504-2
